# Impact of magnetic field regulation in conjunction with the volumetric repainting technique on the spot positions and beam range in pencil beam scanning proton therapy

**DOI:** 10.1002/acm2.13045

**Published:** 2020-10-15

**Authors:** Suresh Rana, Jaafar Bennouna, Alonso N. Gutierrez, Anatoly B. Rosenfeld

**Affiliations:** ^1^ Department of Radiation Oncology Miami Cancer Institute Baptist Health South Florida Miami FL USA; ^2^ Department of Radiation Oncology Herbert Wertheim College of Medicine Florida International University Miami FL USA; ^3^ Department of Medical Physics The Oklahoma Proton Center Oklahoma City Oklahoma USA; ^4^ Centre for Medical Radiation Physics (CMRP) University of Wollongong Wollongong NSW Australia

**Keywords:** magnetic field regulation, pencil beam scanning, proton energy, proton therapy, spot position, volumetric repainting

## Abstract

**Purpose:**

The objective of this study was to evaluate the impact of the magnetic field regulation in conjunction with the volumetric repainting technique on the spot positions and range in pencil beam scanning proton therapy.

**Methods:**

“Field regulation” — a feature to reduce the switching time between layers by applying a magnetic field setpoint (instead of a current setpoint) has been implemented on the proton beam delivery system at the Miami Cancer Institute. To investigate the impact of field regulation for the volumetric repainting technique, several spot maps were generated with beam delivery sequence in both directions, that is, irradiating from the deepest layer to the most proximal layer (“down” direction) as well as irradiating from the most proximal layer to the deepest layer (“up” direction). Range measurements were performed using a multi‐layer ionization chamber array. Spot positions were measured using two‐dimensional and three‐dimensional scintillation detectors. For range and central‐axis spot position, spot maps were delivered for energies ranging from 70–225 MeV. For off‐axis spot positions, the maps were delivered for high‐, medium, and low‐energies at eight different gantry angles. The results were then compared between the “up” and “down” directions.

**Results:**

The average difference in range for given energy between “up” and “down” directions was 0.0 ± 0.1 mm. The off‐axis spot position results showed that 846/864 of the spots were within ±1 mm, and all off‐axis spot positions were within ±1.2 mm. For spots (n = 126) at the isocenter, the evaluation between “up” and “down” directions for given energy showed the spot position difference within ±0.25 mm. At the nozzle entrance, the average differences in X and Y positions for given energy were 0.0 ± 0.2 mm and −0.0 ± 0.4 mm, respectively. At the nozzle exit, the average differences in X and Y positions for given energy were 0.0 ± 0.1 mm and −0.1 ± 0.1 mm, respectively.

**Conclusion:**

The volumetric repainting technique in magnetic field regulation mode resulted in acceptable spot position and range differences for our beam delivery system. The range differences were found to be within ±1 mm (TG224). For the spot positions (TG224: ±1 mm), the central axis measurements were within ±1 mm, whereas for the off‐axis measurements, 97.9% of the spots were within ±1 mm, and all spots were within ±1.2 mm.

## 1 INTRODUCTION

Pencil beam scanning (PBS) delivery technique has become a preferred method relative to passive scattering methods in proton therapy.^1^, ^2^ Pencil beam scanning technique delivers a single pristine beam at a time. Several studies have pointed out the interplay effect between mobile tumor and the delivery of pencil proton beam therapy^3^, ^4^, ^5^, ^6^, ^7^ and carbon ion therapy.^8^, ^9^, ^10^ A volumetric repainting technique has been proposed as one of the motion management techniques to mitigate the interplay in PBS proton therapy.^6^, ^10^, ^11^, ^12^ Volumetric repainting implies repetitive scanning through the whole target volume.^12^, ^13^ During volumetric repainting, repeated scans are delivered in depth, not in the plane transverse to the beam.^12^, ^13^ The beam delivery sequence for volumetric repainting can take several approaches. For example, the whole target volume is irradiated by delivering the proton beam either from the deepest layer to the most proximal layer (“down” direction) or from the most proximal layer to the deepest layer (“up” direction), or a combination of both. The repeated scans of the entire volume allow the delivery of planned dose to the tumor volume repeatedly, thus providing the statistical averaging of dose heterogeneity.^14^

The volumetric repainting technique can be an attractive option to mitigate the interplay effect, mainly because it is independent of any external hardware that may require cooperation from the patient.^3^ Zhang et al.^3^ highlighted the fact that the performance of repainting technique is highly machine‐specific, since spot positions, dose rate, energy switching time, etc. can have an impact on the delivered dose distributions and interplay effect. As pointed out by Zenklusen et al.,^12^ the repainting technique requires a fast energy switching time. Volumetric repainting can be delivered by repetitive scans in depth with beam delivery sequence in “down” direction only. In this case, for multiple repainting, it will require the beamline to switch from the lowest energy to the highest energy of the treatment plan. Such a big energy step may cause the destabilization of the magnets. Pedroni et al.^15^ observed beam positioning displacements of 1 to 3 mm with big energy steps (of the order of the full energy range). An alternative way of delivering the volumetric plan would be to set the beam delivery sequence in “down” direction followed by “up” direction. This would eliminate the need of switching the beamline from the lowest energy to the highest energy of the treatment plan. Instead, after completing the beam delivery in “down” direction, smaller energy steps can be used for the “up” direction, thus minimizing the risks associated with the destabilization of the magnets in the beamline. This requires a strategy to regulate the current to various magnets in the beamline while overcoming the hysteresis of the magnets.

At Miami Cancer Institute, PBS proton therapy is delivered using a ProteusPLUS proton therapy system with a PBS dedicated nozzle (Ion Beam Applications, Louvain‐la‐Neuve, Belgium). The clinical commissioning of our proton system was based on the beam delivery sequence such that irradiation begins from the deepest layer to the most proximal layer. In this case (“down” direction), beam optics was performed with all the magnets in the beamline regulated in the current (CR) mode. Furthermore, the layer switching time for the “down” direction is about 1 s, but it can be up to 6 s for the “up” direction when operated in CR mode. Hence, due to slower layer switching time for the “up” direction in CR mode, the total beam‐on time will increase if the user wants to deliver a volumetric repainting treatment plan that includes the beam delivery sequences in both the “down” and “up” directions.

**Fig. 1:**
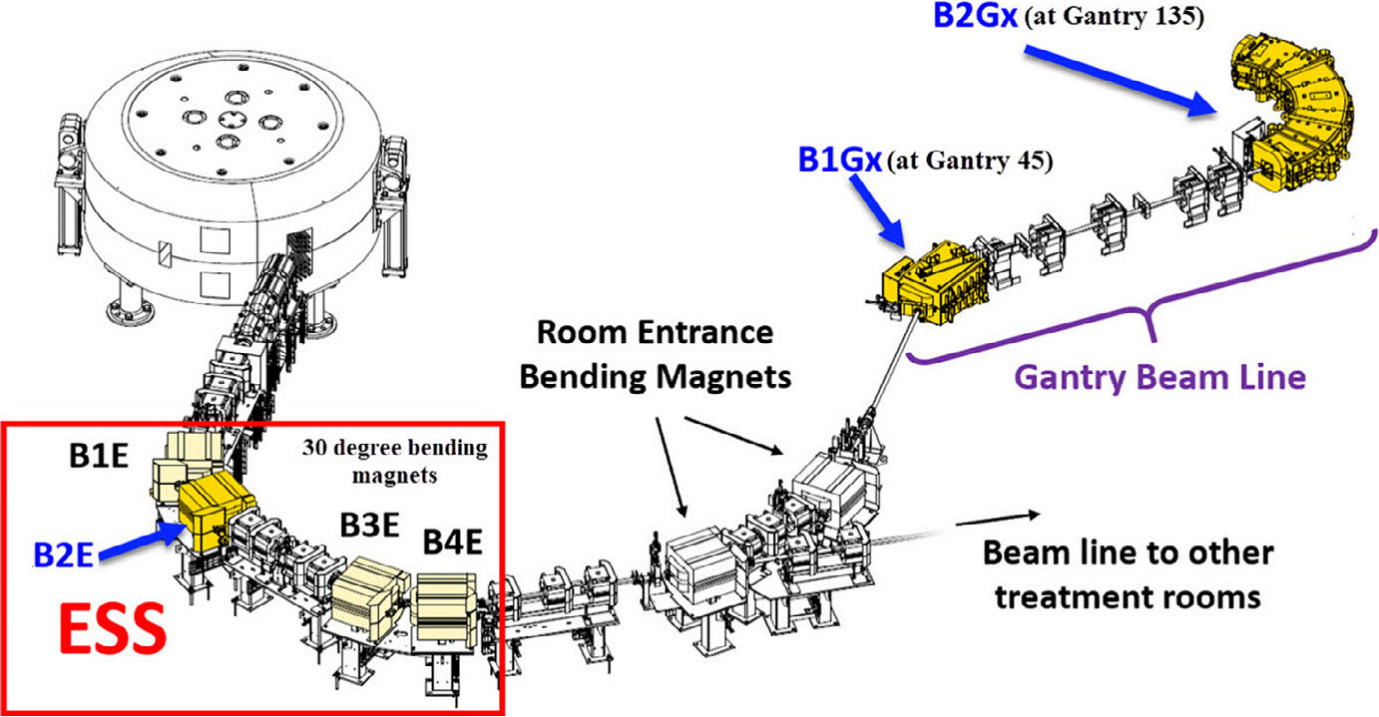
Hall probes mounted inside the 30° bending magnet (B2E) of the energy selection system (ESS) and bending magnets (B1Gx at 45° and B2Gx at 135°) of the gantry.

Recently, our proton therapy vendor has come up with a magnetic field regulation (FR) — a feature to reduce the layer switching time by introducing the Hall probes, which allow measuring the magnetic field in real‐time. Specifically, Hall probes are mounted inside specific groups of magnets in the beamline (Fig. 1). By applying a magnetic field setpoint (instead of a current setpoint) to the specific groups of magnets, there is no requirement of cycling the magnets, except for the first layer of the map, thus reducing the beam stabilization delays and layer switching time in both the “down” and “up” directions. This has decreased the layer switching time for the “up” direction from about 6 s in CR mode to about 1.2–1.3 s in FR mode. The authors believe that the decreased layer switching time for the “up” direction is an important step towards the clinical implementation of the volumetric repainting. However, the use of FR still requires a comprehensive clinical validation.

The proton beam model in our treatment planning system (TPS) is based on the measurements performed in CR instead of FR. In FR mode, all range steps use magnetic field setpoints, which are then linked to the current setpoints by a look up table. Furthermore, FR mode has a Hall probe in the energy selection system (ESS). This brings up the questions — does FR impact the proton beam energy/ranges? Is it necessary to have a new beam model based on range measurements acquired in FR mode? Another critical parameter that could be impacted due to use of FR is the spot position. Psoroulas et al.^11^ investigated that the incorrect magnetic field from the magnets on the gantry can result in spot position errors. The work of Psoroulas et al.^11^ was primarily focused on the CR mode in PSI gantry 2. The addition of Hall probes to the bending magnets in the gantry beam line to measure the magnetic field in real‐time further increases the uncertainty of the spot position errors. If the spots are not delivered at their intended locations during the patient treatment, it will affect the quality of the treatment delivery. Since FR is now available for the clinical use, it brings up additional questions — how does beam delivery direction (“down” vs “up”) impact the spot position errors in FR mode? Should there be a change in the quality assurance (QA) protocol to accommodate the FR and volumetric repainting technique? To the best of our knowledge, these critical questions have not been answered in the literature by providing the experimental data.

In this study, the authors sought to investigate how the combination of FR and volumetric repainting technique impacts the spot positions and range on the PBS beam delivery system. The authors believe that the methodology/technique and results presented herein will serve as the reference for the clinical physicists who are looking to implement FR and volumetric repainting at their proton centers.

## 2 MATERIALS AND METHODS

For our proton therapy system, the clinically available energies range from 70 to 226.5 MeV. Readers are recommended to refer to the published literature^16^, ^17^ for more information on the ProteusPLUS PBS beam delivery system. Figure 1 shows the beam delivery design in FR, which includes a Hall Probe positioned at the entrance/exit of one magnet of the B1234E quadruplet, B1Gx, and B2Gx. B1234E are four 30° bending magnets connected in series. Those are part of the energy selection system (ESS) and contribute to the selection of the correct beam energy. The B1Gx is the bending magnet of the gantry (45°) and the B2Gx is the last bending magnet of the gantry (135°).

### 2.A Range measurements

Range measurements were performed using Giraffe (IBA Dosimetry, Schwarzenbruck, Germany) — a commercial multilayer ionization chamber array. A Giraffe can be used to measure the longitudinal depth‐dose distribution of central‐axis pencil beams. It consists of 180 independent air‐vented, plane‐parallel ionization chambers with a radius of 6.0 cm. More details on Giraffe can be found in the publication by Vai et al.^18^ For range measurements in FR mode, a spot map was generated representing “down” direction followed by “up” direction. Specifically, “down” direction consisted of 32 layers for energies ranging from 225 to 70 MeV at decrements of 5 MeV, whereas the “up” direction consisted of the same number of layers, but the energies ranged from 70 to 225 MeV at increments of 5 MeV. For a comparative purpose, a separate spot map for the “down” direction in CR mode was generated. It included 32 layers for energies ranging from 225 to 70 MeV at decrements of 5 MeV. For both the FR and CR modes, each layer consisted of a single spot at the isocenter. Proton beam was delivered without pausing in between the layers. All measurements were carried out in a movie mode using OmniPro Incline software (IBA Dosimetry, Schwarzenbruck, Germany).

### 2.B Off‐axis spot position measurements

**Fig. 2:**
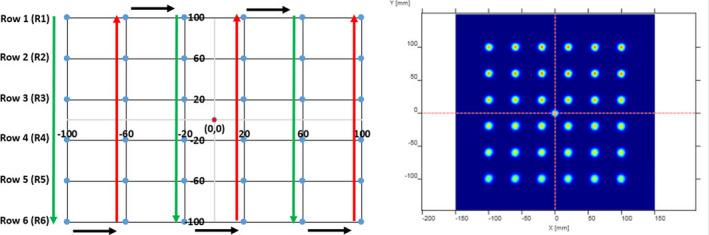
(left) An example of spot map representing beam delivery sequences in “down” and “up” directions; (right) Measured two‐dimensional DICOM image of the spot map.

Off‐axis spot positions measurements were done utilizing the Lynx 2D (IBA Dosimetry, Schwarzenbruck, Germany) — a gadolinium‐based scintillation detector (resolution = 0.5 mm; active surface area = 300 mm × 300 mm).^19^ The Lynx detector was placed at the isocentric plane using the Lynx holder such that the beam is perpendicular to the detector. Three spot maps representing high‐, medium, and low‐energies (Table 1) were generated. Specifically, the first layer of each map included 226.5 MeV at the iscoenter for the reference purpose, whereas the delivery sequence of remaining 36 layers is shown in Figure 2 such that the energy of the spot is in decreasing order from row 1 (R1) to row 6 (R6). For a given row, the energy of all six spots remained the same. All three maps were delivered at eight gantry angles (0°, 45°, 90°, 135°, 180°, 225°, 270°, and 315°) for three groups of energies, as listed in Table 1. Data acquisition was made in a movie mode utilizing myQA software (IBA Dosimetry, Schwarzenbruck, Germany).

**Table 1:** A spot map shown in Fig. 2 is delivered for three groups of energies: high, medium, and low. A spot of 226.5 MeV at the isocenter (0,0) is used as the reference spot.

High energy (MeV) group		Medium energy (MeV) group		Low energy (MeV) group	
Row 1	225	Row 1	160	Row 1	95
Row 2	220	Row 2	155	Row 2	90
Row 3	215	Row 3	150	Row 3	85
Row 4	210	Row 4	145	Row 4	80
Row 5	205	Row 5	140	Row 5	75
Row 6	200	Row 6	135	Row 6	70

### 2.C Central‐axis spot position measurements

Central‐axis spot position measurements were done at gantry angle 0° using XRV‐124 (Logos Systems Int'l, Scotts Valley, CA) — a cone‐shaped scintillator detector. The resolution of the CCD camera using BeamWorksPlus software (Logos Systems Int'l, Scotts Valley, CA) is 1280 × 960 pixels, whereas the BeamWorksPlus software runs at 640 x 480 pixels (binned from 1280 × 960 pixels). The cone has a 140 mm long field of view over 360°, whereas width of the cone varies from 30 to 60 mm.^20^, ^21^ Details on the XRV‐124 can be found in previous publications.^20^, ^21^

For FR measurements, a spot map was generated for the energies ranging from 225 to 70 MeV (“down” direction) followed by 70 to 225 MeV (“up” direction). Each energy layer consisted of a single spot at the isocenter (0, 0), and the energy spacing was 2.5 MeV. For a comparative purpose (FR vs CR), a similar spot map was generated but for beam delivery sequence in the “down” direction for the energies ranging from 225 to 70 MeV at the decrements of 2.5 MeV. Prior to beam delivery, the XRV‐124 detector was aligned to the imaging isocenter by following the procedure described in the literature.^20^, ^21^ Data acquisition was made in a movie mode using BeamWorksPlus software. The beam was delivered without pausing between the layers. The software provides the centricity of the spot in lateral, longitudinal, and vertical directions.^20^, ^21^

Additionally, the beam delivery log files were retrieved to analyze the spot positions at the entrance and exit of the nozzle. Specifically, the first ionization chamber (IC1) provided the X and Y positions at the nozzle entrance, whereas the second ionization chamber (IC2) and third ionization chamber (IC3) provided the Y and X positions, respectively, at the nozzle exit.

## 3 RESULTS

### 3.A Measurements in FR mode

#### 3.A.1 Range measurements

**Fig. 3:**
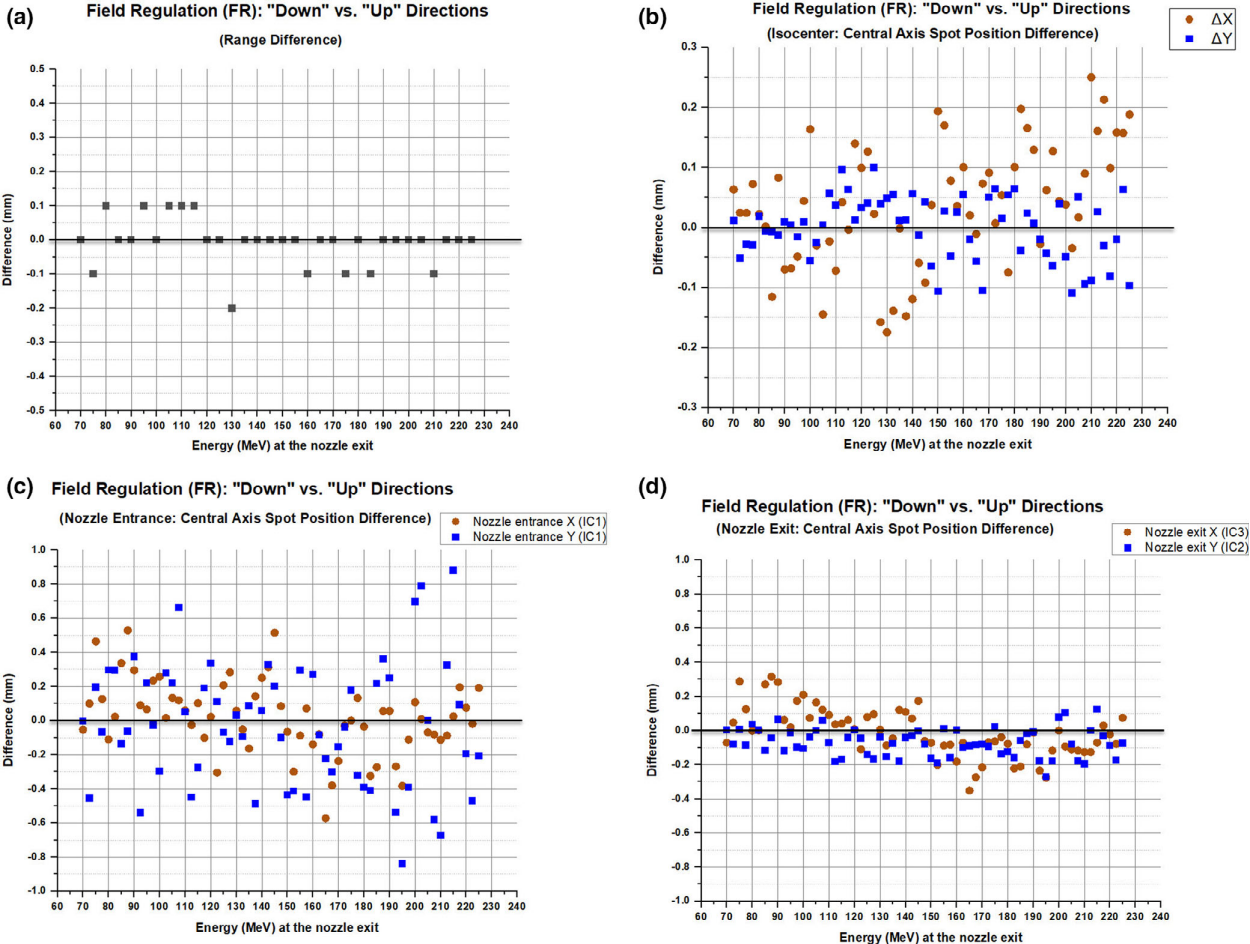
Difference in range and spot positions between the “down” (i.e., distal to proximal) and “up” (proximal to distal) directions for various energies in magnetic field regulation mode.

Figure 3a illustrates the difference in range between the “up” and “down” directions in FR mode. In the current study, the range is defined as the R90 [measured as the penetration depth of the proton beam at 90% point of the normalized percent depth dose (PDD)]. The average difference in R90 for given energy between “up” and “down” directions was 0.0 ± 0.1 mm.

#### 3.A.2 Central‐axis spot position measurements

Figure 3b shows the difference in the position of the central‐axis spots that were delivered to the XRV‐124 scintillation detector at the isocenter. In both the “up” and “down” directions, the positions of the delivered spots (n = 126) were within ±0.5 mm. The spot position evaluation between “up” and “down” directions for given energy also showed the minimal difference (within ±0.25 mm).

Figures 3(c) and 3(d) show the IC1, IC2, and IC3 results, which were retrieved from the log files of the central axis spot position measurements (XRV‐124) as described in Section 2.C. At the nozzle entrance, the average difference in X and Y positions for given energy between “up” and “down” directions was 0.02 ± 0.21 mm (range, −0.57–0.53 mm) and −0.03 ± 0.36 mm (range, −0.84–0.88 mm), respectively. At the nozzle exit, the average difference in X and Y positions for given energy between “up” and “down” directions was −0.01 ± 0.14 mm (range, −0.35–0.32 mm) and −0.07 ± 0.08 mm (range, −0.27–0.12 mm), respectively.

#### 3.A.3 Off‐axis spot position measurements

**Fig. 4:**
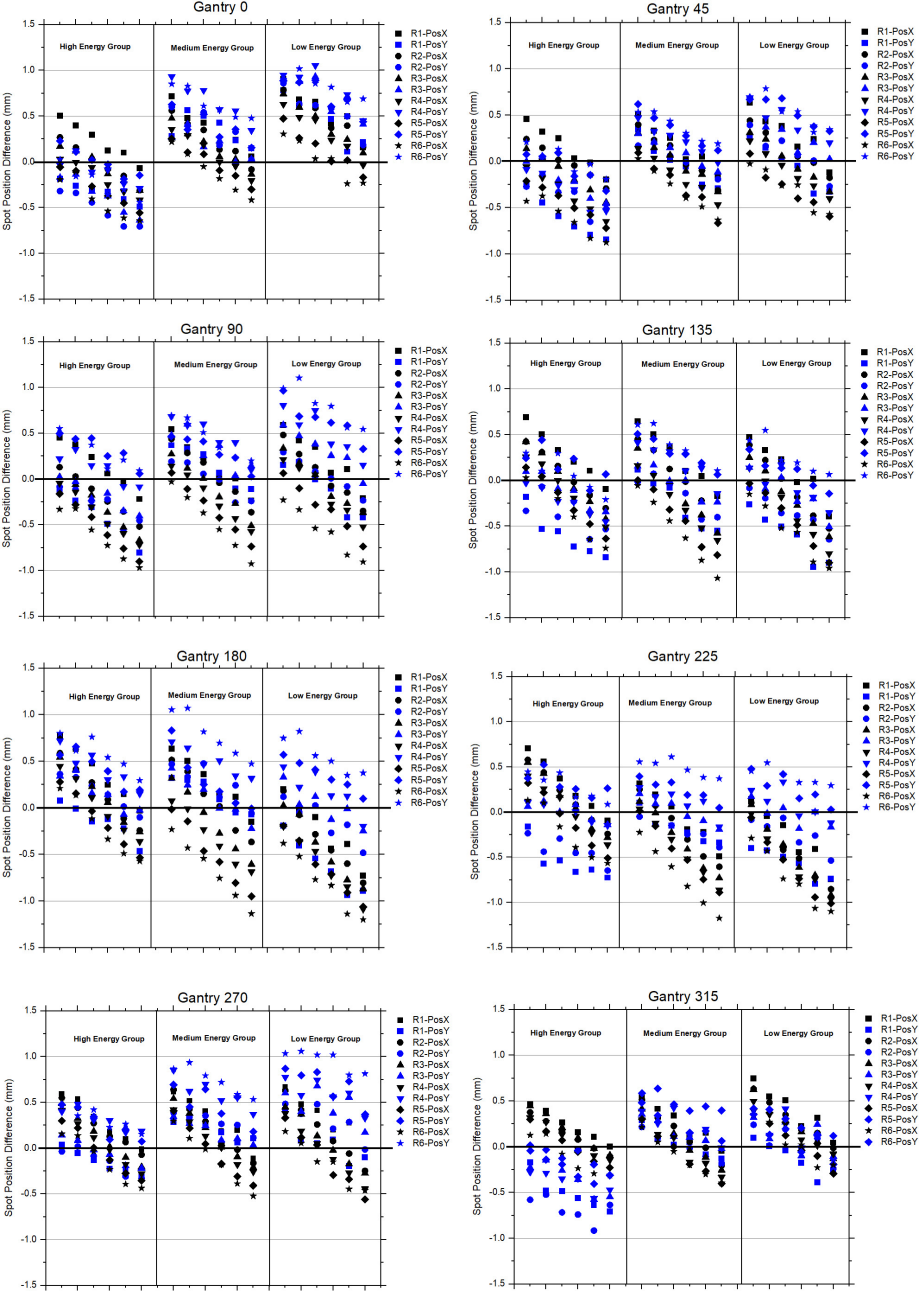
Spot position (X and Y) differences for a spot map shown in Fig. 2 at the gantry angles 0°, 45°, 90°, 135°, 180°, 225°, 270°, and 315°. The spot map was delivered for high‐, medium, and low‐energies as shown in Table 1.

Figure 4 shows the difference in positions of various off‐axis spots that are delivered in a 2D plane (Lynx detector) using the spot map as shown in Fig. 2. The spot at the isocenter was used as the reference spot. The spot maps were delivered for high‐, medium, and low‐energies (Table 1) at eight different gantry angles. A total of 864 off‐axis spots were evaluated to investigate how close these spots can be delivered from their intended locations. The difference in off‐axis spot positions ranged from −0.7 mm to 1.1 mm for gantry 0°, −0.9 mm to 0.8 mm for gantry 45°, −1.0 mm to 1.1 mm for gantry 90°, −1.1 mm to 0.7 mm for gantry 135°, −1.2 mm to 1.1 mm for gantry 180°, −1.2 mm to 0.7 mm for gantry 225°, −0.6 mm to 1.1 mm for gantry 270°, and −0.9 mm to 0.7 mm for gantry 315°. Overall, the off‐axis spot position results demonstrated that 97.9% (846/864) of the spots were within ±1 mm, and all off‐axis spot positions were within ±1.2 mm. Additionally, the evaluation among three different energy groups showed that several spots in the low‐ and medium energy groups had position differences outside ±1 mm.

### 3.B FR mode vs CR mode (“down” direction only)

The comparison between FR and CR modes showed the range difference within ±0.2 mm. The comparison between FR and CR modes showed the selection of either FR or CR had very minimal impact on the spot position. The difference in spot positions ranged from −0.1 to 0.1 mm in both the X‐ and Y‐directions.

## 4 DISCUSSION

In this study, the authors investigated the impact of the FR and volumetric repainting technique on the spot positions and range of proton pencil beams. In our proton therapy system, each magnet has a current setpoint that is linked to the proton beam range through set range tables. However, it is possible to have more than one magnetic field value for a given current setpoint because of the hysteresis of the electromagnets. Hence, for the “down” direction, all magnets are cycled during the set range of the first layer of the delivery map such that the magnetic field is always the same for given proton energy. It is not required to cycle the magnets for the subsequent layers in the “down” direction. However, for the “up” direction in our present site configuration, it is required to cycle the magnets at each set range to ensure the correct magnetic field. The need of cycling the magnets at each set range increases the layer switching time by several seconds in the “up” direction. The placement of Hall probes in the magnets (one in ESS and other two in the gantry beamline) removes the requirement of cycling of the magnets at each set range in the “up” direction, thus reducing layer switching time.

The FR implemented within the IBA ProteusPLUS delivery system allows faster layer switching both in the “down” and “up” directions. The use of FR in the clinical environment is promising, but our proton therapy vendor has made it available after the proton beam model was commissioned. Specifically, beam optics and commissioning have been performed based on the CR mode. Recommissioning the entire proton beam model using FR mode would take a significant amount of time and resources to complete this task. After discussions with the vendor, it was determined that FR mode could potentially affect the proton range and spot positions.

The experimental data presented in the current study demonstrated that the FR resulted in acceptable differences in spot position and range when compared to the CR. The range differences between FR and CR modes were found to be within ±0.2 mm, which is smaller than the range tolerance of ±1 mm recommended by AAPM TG224.^22^ Furthermore, the selection of beam delivery direction (“down” vs “up”) had very minimal impact on the ranges. Hence, if the proton beam model has beam ranges acquired in CR mode, it may not be necessary to obtain new ranges in FR mode for the beam model.

The results from the central axis spot position measurements showed a trend similar to that of the range measurements. The comparison between FR vs CR showed that the positions of the spots are almost identical (±0.1 mm) whether spots are delivered either in FR or CR modes. For a spot map (“down” and “up” directions in FR mode), the position of the central‐axis spots at given energy differed by up to 0.25 mm (Fig. 3). This difference is small compared to the spot position tolerance (±1 mm) recommended by TG224.^22^ However, the off‐axis spot measurements (Fig. 4) showed a larger difference (up to ±1.2 mm) in the spot positions than the central‐axis spot measurements.

As FR is becoming available for the existing and new proton centers, the QA involving FR is not well established yet. Hall probes used in FR may suffer from the variation in temperature, noise, and aging of the detector.^11^ This may result in inaccurate beam delivery due to drift in the range and positions of the spots. Thus, if the FR is implemented clinically, the inclusion of volumetric repainting technique for the routine range and spot positions QA could provide more realistic scenario resembling clinical volumetric repainting beam delivery. It is recommended to use the energy steps of 5 MeV or less to represent the clinical plan.

In this study, the authors did not address the delivery of volumetric repainting maps utilizing patient treatment plans to the detector. This is a limitation of our work. We primarily focused on delivering fields containing several layers, and each layer included a single spot. However, in a real clinical scenario, tumor volume will consist of many spots in each layer depending on the spot spacing and spot width. Currently, we are working with our TPS vendor to design volumetric repainting patient treatment plans. As part of the volumetric repainting project, our next work will include a phantom study that will mimic the tumor motion and quantify the number of volumetric repainting needed to reduce any interplay effect. Despite this limitation, the authors believe that the experimental results from the current study may be useful to the users who are looking to implement FR and volumetric repainting at their proton center. Additionally, the study design and measurement techniques presented in this paper can serve as examples for the experimental validation of a volumetric repainting project.

## 5 CONCLUSION

The combination of FR and volumetric repainting technique resulted in clinically acceptable differences in the spot positions and range for our beam delivery system. The range differences were found to be within ±1 mm (TG224). For the spot positions (TG224: ±1 mm), the central axis measurements were within ±1 mm, whereas for the off‐axis measurements, 97.9% (846/864) of the spots were within ±1 mm, and all spots were within ±1.2 mm.

## CONFLICT OF INTEREST

No conflict of interest.

## References

[1] Chuong M , Badiyan SN , Yam M , et al. Pencil beam scanning versus passively scattered proton therapy for unresectable pancreatic cancer. J Gastrointest Oncol. 2018;9:687–693. 30151265 10.21037/jgo.2018.03.14 PMC6087865

[2] Schreuder AN , Shamblin J . Proton therapy delivery: what is needed in the next ten years? Br J Radiol. 2019;93:20190359. 31692372 10.1259/bjr.20190359 PMC7066946

[3] Zhang Y , Huth I , Wegner M , Weber DC , Lomax AJ . An evaluation of rescanning technique for liver tumour treatments using a commercial PBS proton therapy system. Radiother Oncol. 2016;121:281–287. 27726957 10.1016/j.radonc.2016.09.011

[4] Bert C , Durante M . Motion in radiotherapy: particle therapy. Phys Med Biol. 2011;56:R113–R144. 21775795 10.1088/0031-9155/56/16/R01

[5] Bert C , Grozinger SO , Rietzel E . Quantification of interplay effects of scanned particle beams and moving targets. Phys Med Biol. 2008;53:2253–2265. 18401063 10.1088/0031-9155/53/9/003

[6] Engwall E , Glimelius L , Hynning E . Effectiveness of different rescanning techniques for scanned proton radiotherapy in lung cancer patients. Phys Med Biol. 2018;63:095006. 29616984 10.1088/1361-6560/aabb7b

[7] Seco J , Robertson D , Trofimov A , Paganetti H . Breathing interplay effects during proton beam scanning: simulation and statistical analysis. Phys Med Biol. 2009;54:N283–N294. 19550002 10.1088/0031-9155/54/14/N01

[8] Noda K . Beam delivery method for carbon‐ion radiotherapy with the heavy‐ion medical accelerator in Chiba. Intl J Part Ther. 2016;2:481–489. 10.14338/IJPT-15-00041.1 PMC6871636 31772960

[9] Ebner DK , Tsuji H , Yasuda S , Yamamoto N , Mori S , Kamada T . Respiration‐gated fast‐rescanning carbon‐ion radiotherapy. Jpn J Clin Oncol. 2017;47:80–83. 27677663 10.1093/jjco/hyw144

[10] Kubiak T . Particle therapy of moving targets—the strategies for tumour motion monitoring and moving targets irradiation. Br J Radiol. 2016;89:20150275. 27376637 10.1259/bjr.20150275 PMC5124789

[11] Psoroulas S , Bula C , Actis O , Weber DC , Meer D . A predictive algorithm for spot position corrections after fast energy switching in proton pencil beam scanning. Med Phys. 2018;45:4806–4815. 30273965 10.1002/mp.13217

[12] Zenklusen SM , Pedroni E , Meer D . A study on repainting strategies for treating moderately moving targets with proton pencil beam scanning at the new Gantry 2 at PSI. Phys Med Biol. 2010;55:5103–5121. 20702927 10.1088/0031-9155/55/17/014

[13] Pedroni E . A novel gantry for proton therapy at the Paul Scherrer Institute. AIP Conf Proc. 2001:13–17.

[14] Phillips MH , Pedroni E , Blattmann H , Boehringer T , Coray A , Scheib S . Effects of respiratory motion on dose uniformity with a charged particle scanning method. Phys Med Biol. 1992;37:223–233. 1311106 10.1088/0031-9155/37/1/016

[15] Pedroni E , Meer D , Bula C , Safai S , Zenklusen S . Pencil beam characteristics of the next‐generation proton scanning gantry of PSI: design issues and initial commissioning results. Eur Phys J Plus. 2011;126:66.

[16] Lin L , Ainsley CG , Solberg TD , McDonough JE . Experimental characterization of two‐dimensional spot profiles for two proton pencil beam scanning nozzles. Phys Med Biol. 2014;59:493–504. 24374943 10.1088/0031-9155/59/2/493

[17] Rana S , Bennouna J , Samuel EJJ , Gutierrez AN . Development and long‐term stability of a comprehensive daily QA program for a modern pencil beam scanning (PBS) proton therapy delivery system. J Appl Clin Med Phys. 2019;20:29–44. 10.1002/acm2.12556 PMC6448164 30920146

[18] Vai A , Mirandola A , Magro G , et al. Characterization of a MLIC detector for QA in scanned proton and carbon ion beams. Int J Part Ther. 2019;6:50–59. 31998821 10.14338/IJPT-19-00064.1 PMC6986401

[19] Russo S , Mirandola A , Molinelli S et al Characterization of a commercial scintillation detector for 2‐D dosimetry in scanned proton and carbon ion beams. Phys Med. 2017;34:48–54. 28118950 10.1016/j.ejmp.2017.01.011

[20] Rana S , Samuel EJJ . Feasibility study of utilizing XRV‐124 scintillation detector for quality assurance of spot profile in pencil beam scanning proton therapy. Phys Med. 2019;66:15–20. 31542656 10.1016/j.ejmp.2019.09.078

[21] Cai W , Oesten H , Clasie B , Winey B , Jee KW . Semi‐automated IGRT QA using a cone‐shaped scintillator screen detector for proton pencil beam scanning treatments. Phys Med Biol. 2019;64:085004. 30736026 10.1088/1361-6560/ab056d PMC7448303

[22] Arjomandy B , Taylor P , Ainsley C et al AAPM task group 224: comprehensive proton therapy machine quality assurance. Med Phys. 2019;46:e678–e705. 31125441 10.1002/mp.13622

